# DNA Methylation in Bladder Cancer: Diagnostic and Therapeutic Perspectives—A Narrative Review

**DOI:** 10.3390/ijms26157507

**Published:** 2025-08-03

**Authors:** Dragoş Puia, Marius Ivănuță, Cătălin Pricop

**Affiliations:** 1 Department of Surgery II, “Grigore T Popa” University of Medicine and Pharmacy, 700115 Iasi, Romania; 2 Department of Urology, “Dr. C.I. Parhon” Clinical Hospital, 700503 Iasi, Romania; 3 Center for Morphological and Spectroscopic Analysis of Urinary Stones” Michel Daudon”, 700503 Iasi, Romania

**Keywords:** bladder cancer, DNA methylation, DNA demethylation, epigenetics, tumour suppressor genes, 5-azacytidine, urinary biomarkers, non-invasive diagnosis, inflammation, animal models, gene–environment interaction

## Abstract

Bladder cancer pathogenesis is closely linked to epigenetic alterations, particularly DNA methylation and demethylation processes. Environmental carcinogens and persistent inflammatory stimuli—such as recurrent urinary tract infections—can induce aberrant DNA methylation, altering gene expression profiles and contributing to malignant transformation. This review synthesizes current evidence on the role of DNA methyltransferases (*DNMT1, DNMT3a, DNMT3b*) and the hypermethylation of key tumour suppressor genes, including *A2BP1, NPTX2, SOX11, PENK, NKX6-2, DBC1, MYO3A*, and *CA10*, in bladder cancer. It also evaluates the therapeutic application of DNA-demethylating agents such as 5-azacytidine and highlights the impact of chronic inflammation on epigenetic regulation. Promoter hypermethylation of tumour suppressor genes leads to transcriptional silencing and unchecked cell proliferation. Urine-based DNA methylation assays provide a sensitive and specific method for non-invasive early detection, with single-target approaches offering high diagnostic precision. Animal models are increasingly employed to validate these findings, allowing the study of methylation dynamics and gene–environment interactions in vivo. DNA methylation represents a key epigenetic mechanism in bladder cancer, with significant diagnostic, prognostic, and therapeutic implications. Integration of human and experimental data supports the use of methylation-based biomarkers for early detection and targeted treatment, paving the way for personalized approaches in bladder cancer management.

## 1. Introduction

Bladder cancer remains a significant global health concern, with incidence and prevalence patterns shaped by demographic, environmental, and genetic factors. The disease primarily affects older adults, with most diagnoses occurring in individuals over the age of 65. Epidemiological data consistently show a marked sex disparity, with men being three to four times more likely to develop bladder cancer than women [1]. This imbalance may reflect differences in smoking rates, occupational exposures, and possibly biological susceptibility to carcinogens.

Tobacco smoking is the most prominent modifiable risk factor, accounting for nearly 50% of all bladder cancer cases. Carcinogens present in tobacco smoke are excreted via urine, directly exposing the bladder epithelium to DNA-damaging agents. In addition, occupational exposure to chemicals such as aromatic amines—frequently encountered in the dye, rubber, textile, and leather industries—has been strongly associated with elevated bladder cancer risk [2,3].

Genetic susceptibility also contributes to disease risk. Polymorphisms in genes involved in detoxification pathways (N-acetyltransferase 2 (*NAT2*), Glutathione S-Transferase Mu 1 (*GSTM1*)), DNA repair, and cell cycle regulation may modulate individual vulnerability to bladder carcinogenesis. Besaratinia et al. reported that specific allelic variants may increase bladder cancer risk, particularly among individuals with occupational or lifestyle-related exposure to carcinogens, emphasizing the role of gene–environment interactions in disease development [4].

Early studies in human bladder cancer identified recurrent alterations in both oncogenes and tumour suppressor genes, with epigenetic regulation emerging as a key component in gene expression control during development and disease progression. More recent findings suggest that *c-MYC*—a key oncogenic driver—can both be affected by and actively influence the epigenetic landscape. Specifically, c*-MYC* has been shown to recruit DNA methyltransferases such as *DNMT3A* and modulate chromatin structure, thereby promoting sustained oncogenic transcription [5,6]. 

In bladder cancer specifically, the epigenetic landscape is characterized by widespread promoter hypermethylation, particularly in tumour suppressor genes. The reversal of these modifications through active DNA demethylation has emerged as a promising strategy for restoring normal gene function [7].

DNA methylation has become increasingly recognized as a key regulatory mechanism in the initiation and progression of bladder cancer. By removing methyl groups from Cytosine-phosphate-Guanine dinucleotide (*CpG*), this process reactivates genes that were previously silenced—many of which are involved in tumour suppression, immune response, or cellular differentiation. Its significance extends beyond basic molecular biology, as accumulating evidence points to its value as a potential prognostic indicator in urothelial carcinoma [8,9].

Incorporating both hypermethylation and hypomethylation markers into urine-based assays may improve early detection and enable personalized monitoring of disease recurrence, particularly after TURBT (transurethral resection of bladder tumours) [10]. Several genes, including SRY-Box Transcription Factor 11 (*SOX11*) and Heat Shock Protein Beta-9 (*HSPB9*), have shown consistent methylation differences between bladder cancer patients and healthy controls, with detection methods such as bisulfite pyrosequencing and quantitative methylation-specific PCR (qMSP) proving useful in clinical stratification [2,11].

The ability to analyse large-scale methylation data using AI-driven platforms has accelerated discoveries in this field, enabling the identification of novel epigenetic biomarkers with clinical relevance [7]. These approaches have been particularly useful in bladder cancer, where high heterogeneity complicates traditional genomic analysis. Mapping DNA demethylation profiles alongside patient outcomes is helping to identify epigenetic markers associated with distinct trajectories of bladder cancer progression.

Notably, the prognostic value of demethylation lies in its dynamic nature. Unlike fixed mutations, epigenetic marks are reversible and responsive to environmental and cellular cues. DNA demethylation has been shown to restore the expression of important regulatory genes, which can slow tumour growth and improve the response to standard therapies. In addition, Loo Yau et al. found that hypomethylating agents like decitabine can also enhance the activity of CD8^+^ T cells by triggering a viral mimicry response, leading to better immune-mediated control of tumours. These findings suggest that DNA demethylation may support both direct tumour suppression and improved antitumor immunity [12].

Environmental influences also appear to shape the methylome in bladder cancer. Chronic inflammation—often driven by recurrent urinary tract infections—has been linked to aberrant methylation patterns, suggesting a connection between immune-related stressors and epigenetic instability [8]. Similarly, exposure to carcinogens such as tobacco-derived compounds or industrial chemicals may drive site-specific methylation loss or gain, further emphasizing the gene–environment interplay in disease progression.

From a precision medicine perspective, mapping DNA demethylation patterns could guide more personalized approaches to therapy. Profiling individual epigenetic signatures could enhance patient stratification and inform therapeutic choices, particularly in identifying those most likely to benefit from demethylation-targeted interventions [8]. These personalized approaches are becoming more practical with the growing use of minimally invasive methods for methylation analysis, such as urine-based assays.

In summary, DNA demethylation is emerging as a central player in bladder cancer biology. It not only reflects the epigenetic evolution of the disease but also offers promising diagnostic, prognostic, and therapeutic avenues. As research continues to integrate mechanistic insights with translational applications, demethylation profiling may soon become a routine component of bladder cancer management.

## 2. Approach to Literature Selection

This narrative review was conducted with the aim of synthesizing and contextualizing current knowledge regarding the role of DNA demethylation in bladder cancer pathogenesis and prognosis. The review includes peer-reviewed studies published between 2000 and 2024, focusing on molecular mechanisms, epigenetic regulation, biomarker development, and therapeutic implications associated with DNA demethylation in bladder urothelial carcinoma. Relevant articles were identified through a structured search of the PubMed, Scopus, and Web of Science databases using combinations of the following keywords: bladder cancer, DNA methylation, DNA demethylation, epigenetics, tumour suppressor genes, epigenetic therapy, and urinary biomarkers.

Articles were included if they provided original data or comprehensive reviews related to the demethylation process, its impact on gene expression, and its clinical relevance. Studies were considered eligible for inclusion if they reported original data or comprehensive analyses related to DNA methylation or demethylation in the setting of bladder cancer. Eligible studies included experimental investigations utilizing human tissue specimens, animal models, or validated in vitro systems, provided they contributed to elucidating the underlying biological mechanisms, diagnostic applications, prognostic significance, or therapeutic relevance of epigenetic alterations.

Publications such as editorials, narrative commentaries, conference abstracts lacking accessible data, and non-English language articles were excluded. Where applicable, we have indicated study design, model systems, and sample sizes in the main text to enhance interpretability and context.

This article does not present original experimental data. All information and figures are derived from publicly available peer-reviewed publications cited throughout the manuscript. No new datasets were generated.

The initial literature search retrieved approximately 1.100 articles across PubMed, Scopus, and Web of Science. After removing duplicates and screening titles and abstracts for relevance to bladder cancer and DNA methylation or demethylation, a total of 45 peer-reviewed studies were selected and synthesized in this narrative review.

## 3. Molecular Landscape of DNA Methylation and Demethylation

### 3.1. DNA Methylation Patterns in Bladder Cancer

#### 3.1.1. Active Demethylation Pathways

Active DNA demethylation plays a fundamental role in regulating gene expression by removing methyl groups from DNA, thereby reversing gene silencing mediated by aberrant DNA methylation. The disruption of these pathways in bladder cancer is closely linked to tumorigenesis. Dissecting the enzymatic basis of DNA demethylation may help explain key steps in tumour development and guide the design of targeted therapy.

A primary mechanism of active demethylation involves the Ten-Eleven Translocation (TET) family of enzymes, which catalyse the stepwise oxidation of 5-methylcytosine (5mC) to 5-hydroxymethylcytosine (5hmC), then to 5-formylcytosine (5fC), and finally to 5-carboxylcytosine (5caC). These oxidized derivatives are subsequently recognized and excised by thymine DNA glycosylase (TDG), followed by base excision repair (BER) to restore unmodified cytosines. This pathway is particularly relevant in bladder cancer, where reduced TET activity or impaired 5hmC generation is associated with abnormal DNA methylation patterns and gene silencing [12,13].

In addition to TET enzymes, the AID/APOBEC family of cytidine deaminases also contributes to active demethylation. These enzymes deaminate 5mC to thymine, producing a G: T mismatch that is corrected through the BER pathway, resulting in the restoration of unmethylated cytosine [14]. This mechanism provides an alternative route for the dynamic regulation of gene expression and is increasingly recognized as relevant in the epigenetic remodelling observed in bladder cancer cells [14,15]. 

The interplay between DNA methylation and histone modifications adds an additional layer of complexity. Histone deacetylase inhibitors (HDACi), such as trichostatin A (TSA), have been shown to synergize with DNA-demethylating agents to enhance the reactivation of silenced tumour suppressor genes. Combined epigenetic therapy using 5-aza-dC and *TSA* has been found to significantly increase gene expression in bladder cancer models, highlighting the potential for integrated therapeutic approaches [16].

From a translational perspective, comparative methylation profiling between tumour and adjacent normal tissue has led to the identification of differentially methylated genes that serve as potential biomarkers [17]. Moreover, high-throughput methylation assays applied to urine samples have demonstrated strong potential for non-invasive bladder cancer detection and patient stratification [18].

Therefore, DNA demethylation pathways, particularly those mediated by TET and AID/APOBEC enzymes, are critical to maintaining epigenetic balance and regulating gene expression in bladder cancer. Their disruption contributes to carcinogenesis but also presents opportunities for targeted therapy and molecular diagnostics.

#### 3.1.2. Passive Demethylation Pathways

In addition to active enzymatic mechanisms, passive DNA demethylation plays a pivotal role in shaping the epigenetic landscape of bladder cancer. Unlike active demethylation, passive pathways involve the loss of DNA methylation marks during cell division, typically due to reduced activity or expression of DNMTs, especially DNMT1.

In bladder cancer, passive demethylation can result in the reactivation of tumour suppressor genes that had previously been epigenetically silenced. This phenomenon has I hbeen linked to favourable clinical outcomes in some contexts. For instance, the demethylation of specific prognostic signature genes has been associated with improved survival and reduced tumour aggressiveness. These observations suggest that passive demethylation may serve not only as a molecular marker but also as a modifiable epigenetic process with prognostic value [3].

Moreover, passive demethylation interacts with other layers of epigenetic regulation. The loss of DNA methylation can alter chromatin structure, increase DNA accessibility, and facilitate transcription factor binding. Such changes may enhance the expression of oncogenes or, conversely, derepress tumour suppressor pathways, depending on the genomic context [10,13]. This dual potential makes passive demethylation a complex but essential player in the epigenetic dynamics of bladder tumorigenesis.

One of the most critical consequences of global passive demethylation is its impact on genomic stability. Hypomethylation of repetitive DNA elements, including transposable elements and satellite DNA, has been associated with increased mutation rates, chromosomal rearrangements, and overall genomic instability—hallmarks of cancer progression [17,19]. These alterations contribute to intertumoral heterogeneity and therapy resistance, underscoring the clinical importance of maintaining proper methylation control.

From a translational perspective, assessing passive demethylation patterns may improve the detection and stratification of bladder cancer. Several studies have proposed monitoring gene-specific demethylation status as a non-invasive biomarker for early diagnosis, recurrence risk, and treatment response [20,21].

Therefore, passive DNA demethylation constitutes a critical component of the epigenetic regulation in bladder cancer. Its influence on gene expression, chromatin accessibility, and genome integrity highlights its relevance not only in cancer biology but also in clinical management.

**Figure 1:**
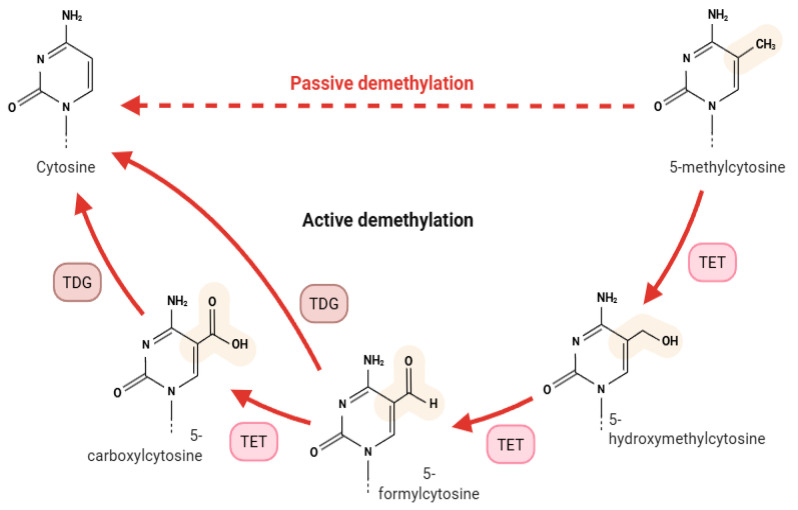
Active and passive mechanisms of DNA demethylation.

In Figure 1 is illustrated both passive and active pathways of cytosine demethylation. Passive demethylation occurs through the replication-dependent dilution of 5mC without maintenance methylation. Active demethylation involves a series of oxidation steps catalysed by TET enzymes, converting 5mC to 5hmC, 5fC, and 5caC. These oxidized bases are subsequently excised by TDG and replaced with unmethylated cytosine via BER.

Passive demethylation occurs through replication-dependent dilution of 5mC due to reduced DNMT1 activity. Active demethylation is mediated by TET enzymes, which oxidize 5mC into 5hm, 5fC, and 5caC, followed by BER.

## 4. DNA Methylation in Bladder Cancer

### 4.1. Hypermethylation of Tumours Suppressor Genes

Hypermethylation of tumour suppressor genes is a critical aspect of the epigenetic landscape in bladder cancer. This process involves the addition of methyl groups to cytosine residues in CpG islands within the promoter regions of tumour suppressor genes, leading to their transcriptional silencing. These epigenetic modifications significantly impact gene expression and contribute to tumorigenesis by disabling the cellular mechanisms that normally inhibit cancer progression.

In bladder cancer, numerous studies have demonstrated the high prevalence of DNA hypermethylation in CpG-enriched regions. For instance, global hypomethylation combined with localized hypermethylation of CpG islands is a common feature in many cancers, including bladder cancer [22]. These aberrant methylation patterns lead to the silencing of critical tumour suppressor genes, facilitating cancer progression and metastasis.

The significance of DNA hypermethylation is further underscored by findings from Zhou et al., who reported that low-grade bladder tumours exhibit fewer changes in methylation sites compared to high-grade and invasive tumours. This correlation suggests that the degree of DNA hypermethylation is associated with tumour aggressiveness and may serve as a prognostic marker [23].

Jiang et al. expanded on this by identifying specific hypermethylation sites within the 3′-untranslated region (3′-UTR) of the *OTX1* gene as useful biomarkers for detection of bladder cancer using urine sediments. Their findings demonstrate the practical applications of DNA methylation profiling in non-invasive diagnostics [15].

Moreover, Kisseljova et al., emphasized the dual role of DNA methylation, explaining that while total DNA demethylation can activate proto-oncogenes, it can also inhibit tumour suppressor genes, thereby increasing the metastatic potential of cancer cells. This duality underscores the complexity of epigenetic regulation in cancer [24].

In addition, Martinez et al. described how DNA methylation interacts dynamically with other epigenetic mechanisms—such as histone modifications and nucleosome positioning—to regulate gene expression. This interplay contributes to the epigenetic heterogeneity and phenotypic identity of tumour cells [25].

The potential of DNA methylation as a non-invasive biomarker is gaining clinical relevance. For example, Koukourikis et al. proposed a panel of hypermethylated genes as a promising diagnostic and risk stratification tool for patients with neurogenic lower urinary tract dysfunction (NLUTD), which could significantly improve early detection and clinical outcomes [26].

Finally, Su et al. demonstrated the effectiveness of urine sediment DNA methylation screening as a non-invasive method for detecting bladder cancer. Their approach focuses on identifying methylation markers present in tumours or urine sediments and is suitable both for initial diagnosis and for monitoring recurrence prior to TURBT [10].

### 4.2. Hypomethylation of Tumours Suppressor Genes

Hypomethylation of oncogenes plays a critical role in the development and progression of bladder cancer. This epigenetic alteration, involving the loss of methyl groups from CpG sites, can lead to the activation of oncogenes.

Kim et al. highlighted the prognostic relevance of CpG methylation dynamics by analysing paired normal and tumour tissue samples. Their study identified differentially methylated CpG markers, with hypomethylation correlating with unfavourable outcomes in bladder cancer patients, thus suggesting these markers as potential prognostic indicators [27].

Further supporting this, Su et al. demonstrated that combining hypomethylation markers—including the transcription factor SOX1, a LINE-1 element, and the epigenetic regulator IRAK3—improved early detection and recurrence monitoring of bladder cancer compared to standard techniques such as cytology and cystoscopy. This emphasizes the potential clinical value of hypomethylation-based diagnostics [10].

Nunes et al. reinforced the importance of aberrant DNA methylation—particularly hypomethylation of oncogenes—as a key feature of bladder carcinogenesis. They emphasized the need for therapeutic strategies targeting these epigenetic alterations to improve patient outcomes [28].

Similarly, Besaratinia et al., examined a four-gene panel in bladder tumours of various stages and grades, finding widespread hypomethylation. This supports the idea that hypomethylation is not limited to advanced disease but may be an early event in tumorigenesis [4].

Thompson et al., discussed the challenges in developing epigenetic therapies for bladder cancer, noting that most existing epigenetic drugs were originally designed for other malignancies and lack specificity for urothelial tumours. By pointing to the role of oncogene hypomethylation, these results suggest it could serve as a rational target for future epigenetic interventions [1].

Consequently, hypomethylation of oncogenes contributes to oncogene activation, promoting tumour progression and correlating with poor prognosis. The identification and clinical validation of hypomethylated CpG markers hold promise for improving diagnosis, monitoring, and therapeutic targeting in bladder cancer.

### 4.3. Impact of DNA Demethylation on Tumour Development and Progression

DNA demethylation plays a crucial role in the development and progression of bladder cancer. This epigenetic process, involving the removal of methyl groups from cytosine residues in DNA, can lead to the activation of proto-oncogenes and the inactivation of tumour suppressor genes, thereby facilitating malignant transformation. One of the major consequences of global DNA demethylation is the hypomethylation of repetitive sequence regions, which contributes to genomic instability, loss of imprinting, and dysregulation of key tumour suppressor pathways [24,29].

The relevance of DNA demethylation as a prognostic indicator in bladder cancer has gained increasing recognition. Although historically overshadowed by studies on hypermethylation, hypomethylation has emerged as a hallmark of several malignancies, including bladder cancer [1]. Studies indicate that the degree of hypomethylation correlates with tumour stage—being more pronounced in advanced tumours (T2–T4) than in early-stage disease (Ta–T1). However, no consistent association has been reported with tumour grade or size [30].

The use of specific methylation markers has proven to be highly effective in distinguishing malignant from non-malignant urothelial tissue. Several studies demonstrate that such markers can be used to detect early-stage, minimal residual, and recurrent bladder tumours with greater sensitivity than standard methods like urine cytology or FISH [31]. Notably, incorporating these methylation-based markers into clinical diagnostic assays may significantly improve the detection of low-grade tumours, which are often missed by conventional screening [18].

Furthermore, the loss of function of epigenetic regulators, such as KDM6A, can induce secondary demethylation changes that contribute to tumour progression. In this context, targeting the gain-of-function effects of such mutations represents a promising therapeutic avenue for KDM6A-mutant bladder cancers [32].

Therefore, DNA demethylation significantly impacts the initiation and progression of bladder cancer by disrupting gene regulation and promoting tumorigenic phenotypes. A more refined understanding of these epigenetic mechanisms is critical for enhancing diagnostic accuracy and uncovering new therapeutic targets, with the ultimate goal of improving clinical outcomes in bladder cancer.

### 4.4. Clinical Implications of DNA Methylation Changes

DNA methylation is a key epigenetic mechanism regulating gene expression and chromatin structure. In bladder cancer, aberrant DNA methylation patterns—including both hypermethylation and hypomethylation—are frequently observed and have substantial clinical relevance. These alterations affect tumour behaviour and can serve as biomarkers for diagnosis, prognosis, and therapy selection.

Studies comparing tumour and normal bladder tissues have identified distinct methylation profiles. Ramakrishnan et al. demonstrated that specific signalling pathways are dysregulated due to methylation changes, suggesting targets for therapeutic intervention [33]. Similarly, Xu et al. reported that bladder cancer tissues exhibit significantly higher rates of demethylation compared to normal urothelium, supporting the role of hypomethylation as a diagnostic biomarker [30].

Urine-based DNA methylation assays represent a promising non-invasive diagnostic tool. Ruan et al. developed a quantitative PCR-based assay using a panel of 22 methylation markers [18]. This method showed high sensitivity for bladder cancer detection, offering advantages over conventional techniques such as cytology and cystoscopy. Piatti et al. confirmed that urine-based methylation markers improve early detection and patient compliance [13].

In addition to diagnostic value, DNA methylation has therapeutic implications. Loss of function of *KDM6A*, a frequently mutated epigenetic regulator in bladder cancer, triggers downstream epigenetic alterations that promote tumour progression. Qiu et al. proposed that targeting these methylation-mediated effects may benefit patients with *KDM6A*-mutant tumours [30]. Furthermore, hypermethylation of tumour suppressor genes, such as *CDKN2A* (*p16*), is a recurrent event in bladder cancer. Jung et al. highlighted the role of DNMT is in restoring gene function and enhancing the effectiveness of cancer therapies [34].

Epigenetic factors may also influence therapeutic response. Singh et al. reviewed the impact of methylation changes on drug resistance and immune evasion, emphasizing the need for integration of epigenetic profiling into bladder cancer management strategies [8].

This direction aligns with broader efforts to identify urinary biomarkers across urological malignancies. For example, the utility of Kidney Injury Molecule-1 (KIM-1) has been explored as a promising biomarker in renal cancer, further supporting the potential of urine-based molecular assays in oncological diagnostics [35].

In Table 1, we summarize the key DNA methylation markers identified in bladder cancer, including their methylation status, detection methods, and current clinical relevance as derived from recent studies.

Taken together, current evidence highlights the pivotal role of DNA methylation alterations in the clinical landscape of bladder cancer. Aberrant methylation patterns, including promoter hypermethylation and global hypomethylation, are involved in tumour initiation, progression, and therapeutic response. Advances in non-invasive assays, particularly urine-based methylation tests, demonstrate strong potential for improving early detection and surveillance strategies. Furthermore, integrating DNA methylation markers into clinical decision-making may facilitate patient stratification and guide targeted interventions. This approach resonates with broader efforts to personalize urological care across different clinical contexts. For instance, longitudinal monitoring strategies have been applied in patients with an acquired solitary kidney post-nephrectomy, highlighting the importance of individualized follow-up in urologic oncology [39]. As such, epigenetic profiling represents a promising avenue for enhancing the precision and effectiveness of bladder cancer management.

## 5. Therapeutic Applications and Clinical Studies

### 5.1. Potential for Reversing Aberrant Methylation Patterns

The potential to reverse aberrant DNA methylation patterns in bladder cancer offers significant opportunities for improving disease management. DNA methylation, a key epigenetic mechanism regulating gene expression, is frequently dysregulated in bladder tumours and contributes to malignant transformation and progression [40]. Promoter hypermethylation of tumour suppressor genes leads to their transcriptional silencing, which may be therapeutically reversible [26].

One approach to restoring normal gene expression involves the use of DNA-demethylating agents. These compounds can reactivate silenced genes by removing methyl groups from CpG-rich regions. For example, reactivation of NOTCH1 expression following demethylation of its promoter and enhancer regions has been demonstrated in bladder cancer cells, highlighting the functional relevance of this strategy [33].

In addition to therapeutic relevance, aberrant DNA methylation also serves as a non-invasive diagnostic biomarker. Detection of hypermethylated DNA fragments in urine has shown promise in identifying early-stage bladder tumours. Specific genomic regions frequently methylated in cancer tissue, but not in normal epithelia, provide high diagnostic specificity and sensitivity [41].

Epigenetic profiling has also revealed prognostic utility. Differentially methylated CpG sites distinguish between tumour and non-tumour samples and correlate with clinical outcomes, offering potential for stratifying patients and guiding treatment decisions [14]. Furthermore, genome-wide analyses demonstrate that DNA methylation is inversely correlated with mRNA expression for several tumour-relevant genes, suggesting functional silencing via promoter methylation [33].

Therapeutically, DNA-demethylating agents may be integrated with existing treatments such as chemotherapy or immunotherapy to improve response rates. Preclinical data suggest that epigenetic reprogramming sensitizes bladder cancer cells to conventional therapies and may overcome resistance mechanisms [23,33].

Such insights reinforce the therapeutic relevance of epigenetic modulation in bladder cancer and highlight the growing need to further explore the mechanisms and clinical applications of DNA demethylation. The growing body of epigenetic evidence holds considerable promise for refining therapeutic strategies and enabling more personalized care.

### 5.2. Demethylating Agents and Their Mechanisms of Action

#### 5.2.1. FDA-Approved Demethylating Agents

Demethylating agents approved by the U.S. Food and Drug Administration (FDA) have shown significant promise in the treatment of multiple cancers, including bladder cancer. These compounds primarily function by inhibiting DNMTs, thereby reversing aberrant hypermethylation and restoring the expression of silenced tumour suppressor genes.

**Figure 2:**
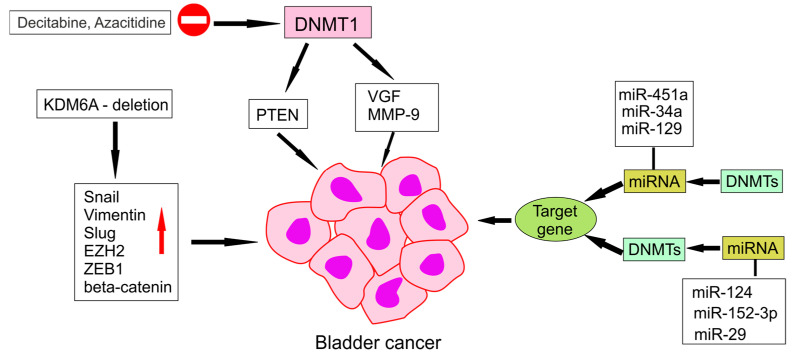
Mechanism of action of DNA hypomethylating agents in cancer. Inhibition of DNMT1 by agents such as decitabine and azacitidine leads to re-expression of silenced tumour suppressor genes. The schematic also highlights regulatory feedback with miRNAs and downstream antitumor effects.

One of the most established agents is 5-azacytidine (5-aza), a nucleoside analogue that incorporates into DNA and RNA and inhibits DNMT activity. This results in genome-wide hypomethylation, reactivation of gene expression, and improved overall survival in patients, although clinical responses may occur without measurable tumour regression. Similarly, decitabine (5-aza-2′-deoxycytidine, DAC) integrates into DNA and selectively inhibits DNMTs, leading to re-expression of epigenetically silenced genes. This mechanism is illustrated in Figure 2, which summarizes the effects of DNMT1 inhibition by agents such as decitabine and azacitidine, including the reactivation of tumour suppressor genes and miRNA-mediated feedback.

A more potent analogue, Aza-T-dCyd, exhibits enhanced DNMT1 inhibition, stronger cytotoxicity, and increased DNA damage compared to both azacitidine and decitabine. Its improved efficacy is attributed to the incorporation of a 4-thio group into its structure. The importance of DNMT1 in mediating these effects has been demonstrated by experiments in which DNMT1 deletion significantly reduced the growth-inhibitory and cytotoxic effects of demethylating agents [42].

In addition to nucleoside analogues, other FDA-approved compounds such as procaine and procainamide—derivatives of 4-aminobenzoic acid—have demonstrated demethylating properties. These agents induce global DNA hypomethylation and inhibit cancer cell proliferation. Notably, procaine reduces DNMT1 and DNMT3a activity without affecting their expression, suggesting a post-translational mechanism of inhibition [28].

Both pharmacological and genetic studies support the anticancer potential of DNMT1 inhibition. Antisense oligonucleotides targeting DNMT1, as well as experiments using DNMT1-deficient mice, have confirmed the enzyme’s central role in maintaining methylation patterns in cancer cells [43].

Emerging data suggest that demethylating agents may also exert immunomodulatory effects. Yau et al. [12] reported that these compounds can reverse de novo methylation programmes associated with T cell exhaustion, leading to enhanced activation of CD8^+^ T cells. This dual mechanism—combining direct tumour suppression with immune activation—positions demethylating agents as promising candidates for immunotherapy.

In this context, combining demethylating agents with immune checkpoint inhibitors (ICIs) represents an attractive strategy. Epigenetic modulation can reverse immune evasion mechanisms within the tumour microenvironment, potentially enhancing the efficacy of ICIs and improving clinical outcomes [12].

Overall, FDA-approved demethylating agents such as 5-aza, decitabine, Aza-T-dCyd, procaine, and procainamide exhibit potent anticancer effects through DNMT inhibition and DNA hypomethylation. Their ability to both reactivates silenced tumour suppressor genes and enhance immune responses underscores their potential as components of combination therapy strategies in bladder cancer and beyond.

#### 5.2.2. Experimental Demethylating Compounds

Experimental DNA-demethylating compounds have attracted considerable interest in bladder cancer research due to their potential to reverse aberrant methylation patterns frequently implicated in tumour initiation and progression. These compounds aim to reactivate silenced tumour suppressor genes and restore normal gene expression programmes in cancer cells.

A major obstacle in the development of demethylating agents is achieving selectivity for malignant urothelial cells while minimizing off-target toxicity in normal tissues. Although several in vitro and in vivo studies have demonstrated promising effects—including cytotoxicity in chemoresistant bladder cancer models—these results have not translated consistently into clinical benefit. A key limitation remains the lack of tumour-specific selectivity, which contributes to dose-limiting toxicity and suboptimal efficacy in human trials [1].

The role of mutations in epigenetic regulators affecting DNA methylation remains underexplored but may hold the key to predicting therapy response. These mutations affect multiple regulatory pathways involved in cell identity, DNA repair, and immune signalling. The identification of methylation-driven differentially expressed genes *(MeDEGs*) has shown potential in uncovering novel therapeutic targets. For example, integrative analyses have revealed hub *MeDEGs* that may serve as candidate biomarkers for guiding treatment decisions in bladder cancer [23].

Mutations in chromatin-modifying genes, such as *KDM6A*, have emerged as important determinants of treatment response. *KDM6A* loss influences epigenetic regulation of DNA repair and metabolic reprogramming and may impact responsiveness to both chemotherapy and immune checkpoint inhibitors (ICIs). Its mutation status could thus inform patient stratification and support the development of personalized epigenetic treatment algorithms [32].

From a diagnostic perspective, DNA methylation-based biomarkers detected in urine samples offer a non-invasive alternative for early detection and surveillance. A systematic review and meta-analysis confirmed the diagnostic accuracy of urinary methylation markers for both primary and recurrent bladder cancer [21]. Notably, methylation of genes such as *TWIST1* and *NID2* has demonstrated high sensitivity and specificity, supporting the clinical utility of urine-based epigenetic tests [44,45].

Beyond diagnosis, the analysis of DNA methylation patterns can inform risk assessment and disease monitoring. Transcriptional shifts in muscle- and neuron-associated genes, which correlate with tumour invasiveness, have been observed early in the disease course, suggesting that methylation profiling may also have prognostic value [46].

Altogether, the continued investigation of experimental demethylating agents and the identification of epigenetic biomarkers represent promising avenues for precision oncology in bladder cancer. By targeting key regulators of methylation and leveraging tumour-specific epigenetic signatures, these approaches may lead to more effective, individualized treatment strategies.

#### 5.2.3. Challenges and Limitations in Therapy Development

One of the most critical obstacles is the inherent heterogeneity of bladder cancer, which encompasses diverse genetic and epigenetic alterations. Inter-patient variability, along with intertumoral differences in DNA methylation patterns, complicates the identification of universal therapeutic targets and contributes to inconsistent treatment responses [2,14].

Another limitation is the incomplete understanding of the biological mechanisms by which DNA demethylation contributes to bladder cancer development and progression. Although aberrant demethylation may activate oncogenes or disrupt tumour suppressor gene function, the downstream pathways and cellular contexts involved remain only partially elucidated [1,4,46].

The non-specific nature of current demethylating agents further complicates therapy development. Because DNA demethylation is a global process, these agents may inadvertently alter the methylation status of genes unrelated to cancer, leading to off-target effects and potential toxicity [20,47,48].

Additionally, DNA methylation is dynamic and reversible, with its regulation influenced by factors within the tumour microenvironment. As a result, the therapeutic effects of demethylating agents may be transient, and tumour cells may adapt through compensatory mechanisms, thereby reducing treatment efficacy over time [1,20,46].

Translating DNA demethylation strategies into clinical settings also involves significant logistical and regulatory challenges. Clinical trials for epigenetic therapies require careful design, including optimized dosing schedules, biomarker-guided patient selection, and well-defined clinical endpoints. Moreover, regulatory approval for novel epigenetic drugs may be delayed by stringent evaluation processes. The integration of demethylating agents into existing treatment regimens introduces additional complexity. Bladder cancer is often treated using multimodal approaches, including surgery, chemotherapy, and immunotherapy. Determining optimal sequencing, dosing, and combination strategies for demethylating drugs remains an active area of investigation. Potential drug–drug interactions and cumulative toxicity must also be carefully considered [10,41]. Despite their therapeutic potential, DNA demethylation-based strategies face important limitations related to disease heterogeneity, mechanistic uncertainty, off-target toxicity, epigenetic plasticity, and translational hurdles. Overcoming these barriers will require a coordinated effort to develop more selective compounds, improve mechanistic understanding, and design clinical trials that reflect the complexity of bladder cancer biology and treatment.

To better illustrate the distinct molecular features and biological implications of DNA demethylation in bladder cancer, Table 2 provides a comparative summary of the active and passive demethylation pathways. This overview highlights their respective enzymatic mechanisms, downstream effects on gene expression, and potential clinical relevance, offering a clearer understanding of how these processes contribute to bladder tumorigenesis.

### 5.3. Prognostic Value of DNA Demethylation in Bladder Cancer

The identification of prognostic biomarkers through DNA demethylation studies has attracted increasing interest due to its potential to refine diagnostic accuracy and guide therapeutic decision-making in bladder cancer. One of the most prevalent epigenetic alterations in malignancies, including urothelial carcinoma, is DNA hypermethylation within promoter regions. This aberrant methylation frequently leads to silencing of tumour suppressor genes, thereby promoting tumorigenesis [41].

In mammalian genomes, DNA methylation predominantly occurs at CpG dinucleotides, where cytosines on both DNA strands are commonly methylated. This process plays a critical role in regulating gene expression across various biological contexts such as development, ageing, and carcinogenesis [47]. Consequently, the discovery of bladder cancer-specific methylation patterns offers a valuable opportunity to develop prognostic and diagnostic biomarkers.

Recent advances in computational approaches have enabled the selection of minimal yet robust panels of methylation markers with high sensitivity and specificity. Several studies have identified such panels by extrapolating from methylation data in other cancers and adapting algorithms for bladder cancer profiling [2]. For instance, longitudinal assessments of DNA methylation in urine sediments from post-resection patients have demonstrated that aberrant methylation of specific loci correlates with tumour recurrence, highlighting their prognostic utility [10].

In addition to classical tumour suppressors, epigenetic regulation of genes involved in muscle differentiation and neuronal signalling has emerged as an early feature of bladder tumorigenesis. As noted by Su et al., alterations in these pathways may contribute to tumour invasiveness and progression, suggesting that early-stage biomarkers could include genes linked to these developmental processes [46]. On this basis, methylation profiling emerges as a valuable tool for both early diagnosis and the design of targeted therapeutic strategies.

Another promising avenue involves the reactivation of epigenetically silenced microRNAs (miRNAs) through pharmacologic agents targeting both DNA methylation and histone modification pathways. The restoration of tumour-suppressive miRNAs has shown therapeutic potential in preclinical models of bladder cancer, offering a layered epigenetic approach to treatment [4].

The potential of urinary biomarkers is not limited to methylation-based assays. Previous studies have explored proteins such as KIM-1 in renal malignancies, demonstrating the clinical feasibility and prognostic value of urine-based molecular detection strategies [35,49]. These findings support the broader application of non-invasive biomarkers in urologic oncology, including bladder cancer, and highlight the translational relevance of integrating such tools into routine clinical workflows.

Given the multifactorial aetiology of bladder cancer—shaped by both genetic predisposition and environmental influences—a comprehensive grasp of its epigenetic architecture is imperative. A growing body of evidence points to DNA methylation abnormalities as key contributors to tumour initiation, progression, and recurrence, reinforcing the clinical relevance of demethylation-driven biomarker discovery [22].

Taken together, these demethylation studies provide a powerful framework for the identification of prognostic biomarkers in bladder cancer. By elucidating specific epigenetic signatures associated with disease course and therapeutic response, these investigations have laid the groundwork for translational applications. Increasingly, such insights are being tested and refined in clinical trials, where DNA methylation markers are evaluated for their diagnostic performance, prognostic value, and therapeutic utility—topics that are explored in the following section.

### 5.4. Clinical Trials and Studies Focusing on DNA Methylation Markers

#### 5.4.1. Clinical Evidence and Translational Insights into DNA Demethylation in Bladder Cancer

In recent years, several clinical trials have investigated the diagnostic and therapeutic utility of DNA methylation markers in bladder cancer. These studies have aimed to validate methylation-based biomarkers identified in preclinical research and to assess their performance in real-world clinical settings. Completed trials have examined the feasibility of incorporating methylation assays into non-invasive diagnostic protocols, evaluated their prognostic accuracy, and explored the efficacy of demethylating agents in epigenetic therapy.

Completed clinical trials have provided significant insights into the role of DNA demethylation in bladder cancer. These studies have explored various aspects of DNA methylation and its potential as a prognostic marker, contributing substantially to the understanding of epigenetic mechanisms in tumour progression and recurrence.

One of the key findings from clinical research is the identification of DNA demethylation as a critical factor in bladder cancer development. Laranjeira et al. demonstrated that agents targeting DNA methyltransferases, such as decitabine and azacitidine, can effectively induce DNA demethylation. Their findings provide a rationale for the therapeutic application of these agents in clinical settings [42].

The prognostic value of DNA methylation markers has been underscored by several meta-analyses. Silva-Ferreira et al. systematically extracted and analysed data from multiple studies, focusing on detection methodologies and outcome correlations. They concluded that specific methylation markers serve as reliable indicators of disease progression and recurrence, thereby assisting in predicting patient outcomes [21].

Bošković et al. reported that epigenetic profiling of tumour tissues reveals accumulated methylation changes, particularly in advanced stages of bladder cancer. While these late-stage alterations may obscure early driver events, their study emphasizes the diagnostic potential of capturing early epigenetic disruptions through DNA methylation analysis [46]. In another comprehensive review, Thompson et al. evaluated the status of epigenetic therapies in solid tumours and emphasized their established efficacy in hematologic malignancies. The authors proposed that similar therapeutic strategies targeting DNA methylation abnormalities could be translated into the management of bladder cancer [1].

The therapeutic implications of targeting epigenetically dysregulated microRNAs have also been explored. Besaratinia et al. highlighted that restoring tumour-suppressive miRNAs through agents that reverse aberrant DNA methylation may improve treatment responses in bladder cancer [4].

Beyond individual biomarkers, integrative epigenetic frameworks are also being developed. Shivakumar et al. proposed a model that incorporates interactions between DNA methylation and miRNA expression based on TCGA data. Their analysis revealed significant associations with prognosis and suggested a multidimensional approach to understanding epigenetic regulation in urothelial carcinoma [50].

Qiu et al., investigated the role of *KDM6A* loss in reshaping the epigenetic landscape of bladder cancer, showing that this mutation disrupts specific transcription factor circuits rather than inducing widespread epigenomic alterations—offering mechanistic insight into the epigenetically driven transformation of urothelial cells [30]. Similarly, Zhang emphasized the therapeutic potential of targeting DNA methylation due to its strong association with tumorigenesis, suggesting that modulation of methylation dynamics could form the basis of future treatment strategies [32]. Collectively, the results of completed clinical trials and translational studies underscore the clinical relevance of DNA demethylation in bladder cancer. These investigations establish a foundation for biomarker-guided diagnosis and personalized therapeutic approaches, advancing the precision and effectiveness of bladder cancer care.

#### 5.4.2. Ongoing Clinical Trials

One major focus is the therapeutic application of DNA-demethylating agents such as DAC. Preclinical studies have shown that low, non-cytotoxic doses of DAC induce targeted demethylation and upregulate tumour-suppressive genes such as *NOTCH1*, thereby restoring transcriptional activity and impairing tumour progression [33]. The results point to demethylation-driven epigenetic reprogramming as a means of reversing aberrant gene silencing and rebalancing cellular behaviour in bladder cancer.

In parallel, current trials are evaluating the utility of epigenetically dysregulated miRNAs as biomarkers for disease detection, prognosis, and therapeutic response. Aberrant methylation at miRNA promoter regions and CpG islands has been widely reported in bladder cancer. Ongoing studies are investigating whether restoring the expression of these silenced miRNAs—using agents that simultaneously target DNA methylation and histone modifications—could serve as an effective therapeutic strategy.

Another promising direction involves targeting enzymes directly responsible for methylation maintenance. For instance, the methyltransferase *DNMT1* has emerged as a potential oncogenic driver. Evidence indicates that silencing *DNMT1* expression reduces tumour cell proliferation and migration, while its overexpression promotes malignant behaviour [1]. Accordingly, *DNMT1* inhibition is being explored in clinical trials as a means of disrupting key epigenetic pathways in bladder tumorigenesis.

Ongoing clinical research also incorporates system-level frameworks to understand the interplay between DNA methylation and transcriptomic alterations. Shivakumar et al. proposed an integrative model to detect epigenetic interactions between methylation and miRNA expression, demonstrating significant associations with patient prognosis. This integrative approach is now being translated into clinical settings, with the goal of improving prognostic precision and informing targeted interventions [50].

Emerging evidence also highlights the role of immune modulation through epigenetic regulation. Demethylation of regulatory elements within the IFN-γ locus has been implicated in T cell functional reprogramming, with histone modifiers such as EZH2 also playing a role [25]. Clinical trials are now examining how targeting these regulatory axes could enhance responsiveness to immunotherapies, potentially overcoming resistance in bladder cancer.

In summary, ongoing clinical trials centred on DNA demethylation are advancing our understanding of epigenetic mechanisms in bladder cancer. Through therapeutic demethylation, miRNA reactivation, DNMT inhibition, integrative biomarker modelling, and epigenetic immunomodulation, these efforts seek to translate fundamental discoveries into more effective, individualized patient care. To provide a clearer view of the current landscape, Table 3 presents a selection of relevant clinical trials, highlighting their objectives, approaches, and outcomes where available. This synthesis helps illustrate how demethylation-based strategies are gradually finding their place in clinical oncology.

## 6. Limitations and Future Directions

Despite notable progress in identifying epigenetic biomarkers for bladder cancer, several challenges remain before their widespread clinical adoption. Urine-based methylation assays, although promising as non-invasive tools, still face limitations in terms of diagnostic performance. For instance, studies evaluating the TWIST1 and NID2 methylation panel have reported lower sensitivity in larger, independent cohorts compared to initial findings—sometimes dropping below thresholds acceptable for clinical use. Moreover, the net clinical benefit of these tests appears modest, particularly in scenarios where clinicians have a low tolerance for missed cases. These concerns underline the need for further validation in large, well-characterized populations before such assays can replace or complement cystoscopy in routine practice [51].

Similarly, while hypomethylating agents such as decitabine and azacitidine show potential in reactivating tumour suppressor genes and enhancing immune responses, their therapeutic application in bladder cancer remains limited. Systemic toxicity—especially haematological side effects like myelosuppression—poses a major barrier, particularly in older patients or those with comorbidities. In addition, the risk of off-target demethylation and lack of tumour specificity reduce their safety margin. Future directions may involve more selective epigenetic modulators or targeted delivery approaches, combined with predictive biomarkers to identify those patients most likely to benefit from such interventions.

Elucidating the molecular mechanisms that govern active and passive DNA demethylation in bladder cancer is equally essential. Gaining clearer insight into how these processes regulate gene expression and tumour behaviour may reveal new therapeutic targets. In this context, the clinical relevance of DNMTs remains an area of active and necessary investigation. Combinatorial strategies, such as DNMTi administration with metabolic modulators like tetrahydrouridine, have shown synergistic effects in preclinical models and are currently under clinical evaluation [47].

In parallel, the development and clinical implementation of non-invasive diagnostic assays based on DNA methylation status remain a key research priority. Platforms such as UroMark have shown high sensitivity and specificity in detecting bladder cancer using urinary DNA methylation signatures, offering a promising alternative to cystoscopy for early detection and surveillance [3].

Longitudinal evaluation of therapies targeting DNA methylation machinery is another important frontier. While early-phase trials report encouraging outcomes, the long-term safety, durability of response, and potential resistance mechanisms remain to be fully characterized. Addressing these issues is essential for optimizing treatment regimens and ensuring sustainable clinical benefit [28].

Finally, integrating multi-omics approaches—including genomics, transcriptomics, and epigenomics—will be instrumental in constructing a holistic model of bladder cancer biology. Such integrative analyses can uncover complex regulatory networks involving DNA demethylation and facilitate the identification of robust, clinically actionable biomarkers and therapeutic targets [10].

Future research on DNA demethylation in bladder cancer holds considerable promise for enhancing both our understanding of disease pathophysiology and the development of novel therapeutic strategies. A critical area of focus will be the identification and rigorous validation of demethylation-based biomarkers capable of predicting disease progression and patient outcomes. Prior studies have demonstrated associations between gene-specific hypermethylation and bladder cancer prognosis, suggesting that demethylation profiles may serve as reliable prognostic indicators [14,47].

## 7. Conclusions

DNA demethylation is emerging as a critical component in the epigenetic regulation of bladder cancer, influencing gene expression, tumour behaviour, and clinical outcomes. By reactivating silenced tumour suppressor genes, demethylation contributes not only to improved understanding of disease mechanisms but also to the development of novel prognostic and therapeutic strategies.

Technological advances, including AI-assisted epigenomic profiling and non-invasive detection tools such as urine-based methylation assays, are accelerating the identification of clinically relevant biomarkers. These approaches offer promising avenues for early diagnosis, risk stratification, and personalized treatment planning, particularly when combined with patient-specific methylation signatures.

Continued research in this field is vital to translate current findings into clinical applications. By leveraging demethylation-based biomarkers and therapeutic strategies, the field moves closer to achieving truly individualized care for bladder cancer patients—marked by improved accuracy in diagnosis, responsiveness to treatment, and long-term disease control.

## Figures and Tables

**Table 1:** Key DNA methylation biomarkers associated with bladder cancer. This table summarizes genes found to be hypermethylated in bladder tumours, along with their clinical relevance, detection method, and associated prognostic or diagnostic implications. Abbreviations: qMSP = quantitative methylation-specific PCR.

Author	Gene	Methylation Status	Clinical Relevance	Detection Method
Su [10]	*SOX11*	Hypermethylated	Early detection marker; frequently methylated in non-invasive bladder tumours, aiding in differentiation from benign conditions	Bisulfite pyrosequencing
	*HSPB9*	Hypermethylated	Potential urinary biomarker for early-stage bladder cancer; supports non-invasive diagnosis	Bisulfite pyrosequencing
Jung [34]	*CDKN2A (p16)*	Hypermethylated	Commonly hypermethylated in high-grade tumours; may predict response to DNMT inhibitors	qMSP, literature review
Chung [2]	*NPTX2*	Hypermethylated	Silencing contributes to reduced synaptic activity; potential diagnostic and prognostic marker	qMSP
	*PENK*	Hypermethylated	Methylation correlates with tumour aggressiveness and recurrence risk	qMSP
	*NKX6-2*	Hypermethylated	Frequently silenced in bladder cancer; loss of function linked to decreased apoptotic signalling	qMSP
	*MYO3A*	Hypermethylated	Emerging prognostic biomarker: methylation status associated with tumour subtype differentiation	qMSP
	*CA10*	Hypermethylated	May influence tumour cell motility; associated with adverse histopathological features	qMSP
Ruan [18]	*WIF1*	Reactivation after demethylation	Potential diagnostic value; differential methylation observed between tumour and adjacent normal tissue	In vitro cell line assay
Kandimalla [36]	*MEIS1*	Hypermethylated	Highly methylated in MIBC, poor prognosis	Tissue
	*SYNPO2*	Hypermethylated	Associated with BCG resistance	Tissue
Lin [37]	*APC*	Hypermethylated	Diagnostic marker; high detection rate with FGFR3	Urine/Tissue
	*RASSF1A*	Hypermethylated	Diagnostic/prognostic; high detection with FGFR3	Urine/Tissue
	*SFRP2*	Hypermethylated	High detection accuracy when combined with FGFR3 mutation	Urine/Tissue
Maruyama [38]	*CDH1*	Hypermethylated	Associated with poor survival; independent predictor	Urine/Tissue
	*FHIT*	Hypermethylated	Associated with poor survival	Urine/Tissue

**Table 2:** Comparison between active and passive DNA demethylation mechanisms relevant to bladder cancer. The table outlines key enzymes, molecular pathways, and clinical implications for both demethylation types. TET-mediated oxidation and DNMT1 inhibition are central to active and passive pathways, respectively.

Demethylation Type	Key Enzymes	Mechanism Description	Example Genes Affected	Clinical Relevance	Key Enzymes
Active	TET1/2/3, TDG	5mC → 5hmC/5fC/5caC → BER-mediated replacement	WIF1, CDKN2A	Tumour suppressor reactivation	TET1/2/3, TDG
Passive	DNMT1 inhibition	Failure of maintenance during replication	Prognostic markers (e.g., SOX11)	Associated with tumour progression	DNMT1 inhibition

**Table 3:** Overview of clinical and preclinical studies on DNA demethylation in bladder cancer.

Author	Intervention/Agent	Mechanism	Phase	Outcomes
Laranjeira et al. [42]	Decitabine, Azacitidine	DNMT inhibition	Preclinical	Effective demethylation and tumour inhibition
Ramakrishnan et al. [33]	Low-dose Decitabine	NOTCH1 upregulation	Preclinical	Reversal of gene silencing
Thompson et al. [1]	DNMT1 silencing	Epigenetic therapy	Review	Growth suppression via *DNMT1* inhibition
Qiu et al. [32]	—	KDM6A pathway	Translational	TF network disruption in KDM6A-mutant tumours

## Data Availability

No new data were created or analyzed in this study. Data sharing is not applicable to this article.

## Abbreviations

The following abbreviations are used in this manuscript:

NAT2	N-acetyltransferase 2
GSTM1	Glutathione S-Transferase Mu 1
TURBT	transurethral resection of bladder tumours
SOX11	SRY-Box Transcription Factor 11
HSPB9	Heat Shock Protein Beta-9
CpG	Cytosine-phosphate-Guanine dinucleotide
AI	Artificial Intelligence
TET	Ten-Eleven Translocation
5mC	5-methylcytosine
5hmC	5-hydroxymethylcytosine
5fC	5-formylcytosine
5caC	5-carboxylcytosine
TDG	thymine DNA glycosylase
BER	base excision repair
HDACi	Histone deacetylase inhibitors
TSA	trichostatin A
3′-UTR	3′-untranslated region
NLUTD	neurogenic lower urinary tract dysfunction
PCR	Polymerase Chain Reaction
KIM-1	Kidney Injury Molecule-1
FDA	Food and Drug Administration
DNMTs	DNA methyltransferases
5-aza	5-azacytidine
MeDEGs	methylation-driven differentially expressed
miRNAs	silenced microRNAs
